# Whose well-being? Common conceptions and misconceptions in the enhancement debate

**DOI:** 10.3389/fnsys.2014.00148

**Published:** 2014-08-19

**Authors:** Stephan Schleim

**Affiliations:** ^1^Theory and History of Psychology, Faculty of Behavioral and Social Sciences, Heymans Institute for Psychological Research, University of GroningenGroningen, Netherlands; ^2^Research Center for Neurophilosophy and Ethics of Neurosciences, Munich Center for Neurosciences, Ludwig-Maximilians-University MunichMunich, Germany

**Keywords:** neuroethics, cognitive enhancement, science communication, biological psychiatry, translational science, framing, well-being, world happiness report

For this Research Topic on brain augmentation,^1^ several authors discuss possibilities of brain stimulation (e.g., Duecker et al., 2014), pharmacology (e.g., Lynch et al., 2014), and psychobiological training (e.g., Chapman and Mudar, 2014). According to a definition proposed by ethicists, such procedures are human enhancement if and only if they are a “change in the biology or psychology of a person which increases the chances of leading a good life in the relevant set of circumstances” (Savulescu et al., 2011b, p. 7). Note how this definition describes the individual as malleable and the circumstances as given. The authors continue to explain that something counts as enhancement “so long as it tends to increase a person's well-being” (Savulescu et al., 2011b). Similarly, Nagel emphasizes the notions of happiness, well-being, and improvement in her discussion of the ethical challenges of enhancement and discusses the possibilities and risks related to neuro-technology and psychopharmacology (Nagel, 2014).

These and similar publications identify concepts like improvement or well-being as foundational issues of the enhancement debate. This raises important questions, such as who defines well-being and how to achieve it. In the three following sections, I will discuss the conceptualization of well-being, the framing of enhancement, and the translational promises given in the literature.

## Whose well-being?

The majority of the experimental enhancement literature employs neuropsychological test designs developed to measure the presence of psychological impairment in terms of attention, learning, memory, and the like (for systematic reviews, see Repantis et al., 2010; Smith and Farah, 2011; Bagot and Kaminer, 2014). Referring to this literature in the human enhancement debate is problematic: That these tests can be used to inform clinical decisions does not warrant their usefulness outside the clinics. Higher test scores do not necessarily reflect a happier, more meaningful life in general. Yet, clinical studies are often cited in ethical discussions to debate the benefits and prospects of enhancement for the healthy. This carries the risk of a normative fallacy, namely, the identification of clinical benefit with overall well-being.

This risk is often accompanied by another one, namely, that of a localizational fallacy. It consists in only targeting individuals psychobiologically, not their circumstances. In contrast, established measures such as the *World Happiness Report* which are provided by United Nations institutions measure well-being macroscopically: GDP per capita, social support, healthy life expectancy at birth, freedom to make life choices, generosity, and perceptions of corruption together explain 75.5% of the international variance of happiness rankings in 2012 (Helliwell et al., 2013). It goes without saying that these indices are also based on norms, but not primarily driven by clinical needs, instead broader in scope, and developed by institutions which are representing people at large at least remotely.

An advanced recent proposal consists in the *OECD Guidelines on Measuring Subjective Well-being*, operationalizing subjective well-being as consisting of life satisfaction, affect, and eudaimonic well-being, which in turn consist of three subcategories each, namely, income, health, and work satisfaction; anger, worry, and happiness; competence, autonomy, and meaning and purpose (OECD, 2013). Based on these guidelines, people can create their own *Better Life Index*, prioritizing 11 pre-defined dimensions (such as housing, jobs, education, or safety), and more than 60,000 citizens from OECD countries have so far participated^2^. Using such methods, the risk of a normative fallacy can be minimized, since people can choose their own standards, although ideally they should be able to design the methods, too. The results, including meaningful differences between countries, indicate that human enhancement need not be localized in individual psychobiology, but can also be achieved by socio-political reform.

It turned out, for example, that safety is valued most highly by participants from Japan, income and housing by those in the United States, and education by those in Finland. To assess the relevance of brain stimulation, pharmacology, and psychobiological training for human enhancement, it would be informative to know to what extent these methods can contribute to human well-being broadly understood. If it turned out that the causal link is very remote and speculative, proponents of human enhancement could conclude that socio-political reform is more promising a means than individual psychobiological intervention. In the terms of the definition proposed by Savulescu and colleagues above, this amounts to not changing the subject with respect to the circumstances, but the circumstances with respect to the subject.

## Framing and relevance

Cognitive enhancement has been framed as common by leading scholars in the field who described it as a means “not to get high, but to get higher grades, to provide an edge over their fellow students or to increase in some measurable way their capacity for learning” (Greely et al., 2008, p. 702). Greely and colleagues subsequently stated that almost 7% of students in the US already use stimulants like amphetamine or methylphenidate for cognitive enhancement, with the prevalence reaching 25% on some campuses. In a comment gathering some anecdotal evidence, I pointed out that such framings occur regularly in the ethical literature (Schleim, 2010). This impression is shared by Lucke et al. (2011) who also carried out a media analysis of newspaper articles and found that 94% of the reports mentioning the prevalence of psychopharmacological enhancement described it as common, increasing, or both (Partridge et al., 2011). Actually, 66% of these reports referred to the academic literature as evidence. It goes without saying that this framing of the practice as common and/or increasing lends the topic high urgency.

In the systematic review of prevalence studies in student samples by Smith and Farah, the most comprehensive I know of, the authors conclude that “[a]mong college students, estimates of use vary widely but, taken together, suggest that the practice is commonplace” (Smith and Farah, 2011, p. 717). Referring to this review, Nagel even claims that the usage is increasing (Nagel, 2014). Both claims are difficult to justify, though, with respect to cognitive enhancement: First of all, it is in the eye of the beholder what to count as common. The decision is complicated by the variance in findings, ranging from 1.7 to 34% in studies with more than thousand students (*N* = 12; mean = 9.5%, median = 6.7%). Sometimes the reported figures reflect past month prevalence (*N* = 2; mean = 4.6), sometimes they refer to last year (*N* = 6; mean = 6.7) or even lifetime usage (*N* = 4; mean = 16.1). Secondly, their authors often investigated non-medical use, which allows many different motives for stimulant consumption that do not indicate cognitive enhancement, such as feeling high or losing weight. Smith and Farah summarize that in those surveys addressing motives, study-related answers were dominant but regularly accompanied by recreational/lifestyle choices (Smith and Farah, 2011). However, detailed interviews with consumers at an elite university in the United States suggest that emotional rather than cognitive motives drive non-medical use even for improving studying, since people report feeling better and overcoming motivational problems with stimulants (Vrecko, 2013).

**Figure 1:**
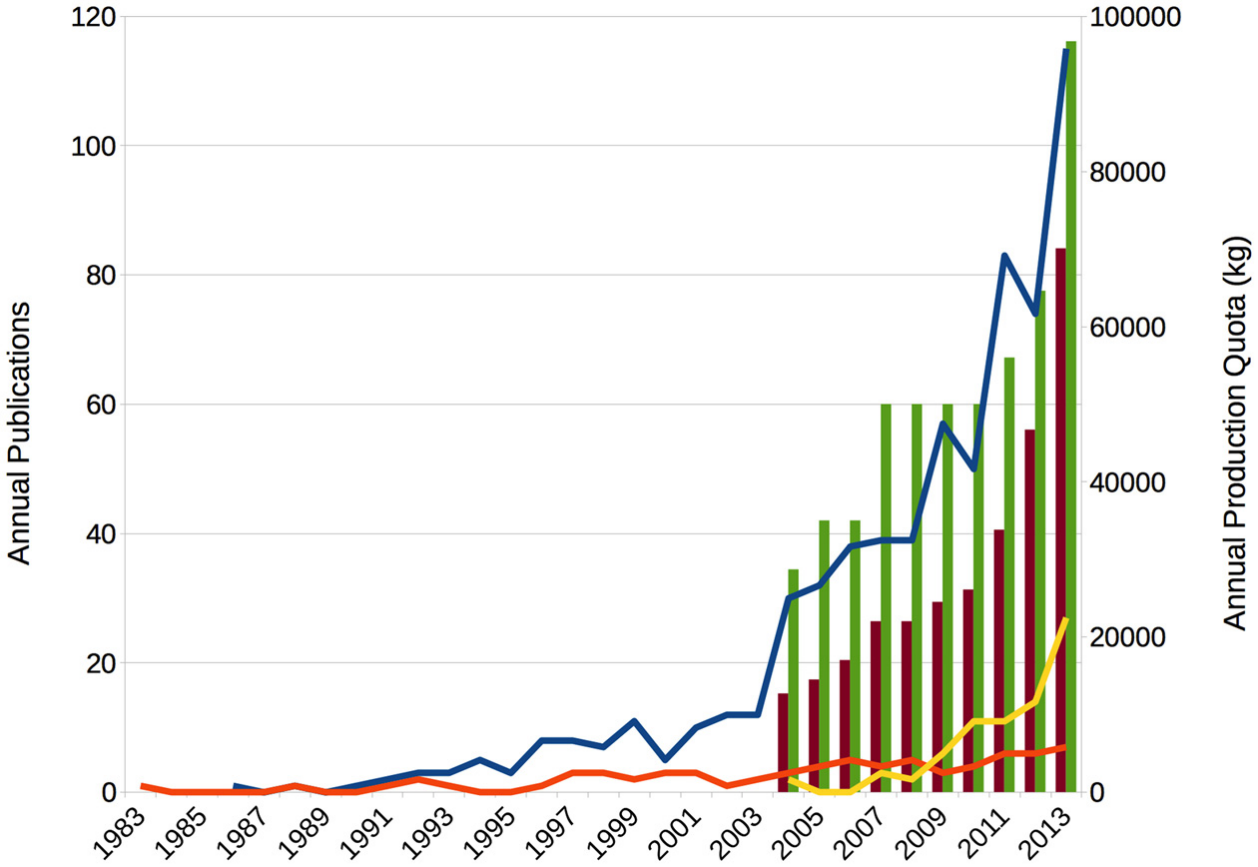
**Stimulant production and enhancement papers increased strongly**. Lines show a steep increase in publications on cognitive enhancement (blue) and neuroenhancement (yellow), but only modestly on mood enhancement (orange). Publication numbers are based on a Web of Science topic search. Bars show a strong increase in production quotas for amphetamine (red) and methylphenidate (green). In the shown 10-year period from 2004 to 2013, the former increased 5.5-fold, the latter 3.4-fold, after quotas had already been increasing in the 1990s (not shown, but see Rasmussen, 2008). Figures based on US Drug Enforcement Agency, October 2, 2013, http://www.deadiversion.usdoj.gov/quotas/quota_history.pdf (accessed May 30, 2014), accumulating amphetamine produced for sale and conversion.

For the time being, framing the relevance as common and non-medical use as cognitive enhancement is therefore, in my view, in contrast to the best available evidence. It is even more problematic to claim that the practice is increasing, because this would require repeated cross-sectional studies of comparable samples under standardized conditions. Yet, even within research groups definitions of inclusion criteria and ways of sampling data often differ. Nevertheless, what has been increasing steeply during the last decades was the production of stimulants like amphetamine and methylphenidate, particularly in the United States, and publications on enhancement (see Figure 1). That the former increase is not reflected in the prevalence studies previously mentioned is most likely due to the concept of non-medical use. Both drugs are controlled prescription stimulants and most epidemiologists as well as ethicists strictly distinguish medical use as treatment from non-medical use as either drug abuse or enhancement.

This framing has wider ramifications for the scientific community: Without the treatment/enhancement distinction, the consumption of stimulants can and has been analyzed by medical sociologists under labels such as medicalization or pharmaceuticalization (Abraham, 2010; Bell and Figert, 2012); and without the claim that enhancement is common or even increasing, the problem appears much less urgent. By framing stimulant consumption as enhancement and common, though, neuroethicists generated a new ethical problem, new prospects and risks, that they subsequently could manage (see also Conrad and De Vries, 2011; Littlefield and Johnson, 2012). Indeed, the steep increase in publications on enhancement topics coincides with the inception of instutionalized neuroethics (Marcus, 2002; Farah, 2012; Figure 1). It thus becomes apparent that both, medical sociologists and neuroethicists, have a conflict of interest in framing stimulant consumption in the competition for research funds and high-impact publications.

## Promises

The abundant literature on enhancement suggests the possibility to increase learning, to feel better, and to become more intelligent by means of brain stimulation, pharmacology, or psychobiological learning (Savulescu et al., 2011a; Farah, 2012; Hildt and Franke, 2013; Nagel, 2014). However, it is also noted that there is much that is not known about the working of stimulants, for example, and that funding of empirical research is difficult because it is not about treatment and therefore outside the purview of disease-oriented schemes and it is too applied for funders of basic science (Smith and Farah, 2011). As mentioned in the section on well-being above, it is furthermore not clear what the goal of the intervention is and whether changing the individual in its circumstances is actually more promising than changing the circumstances for the individual.

However, by analogy with biological psychiatry it is possible to at least engage in informed speculation on what the situation might be like had there been more agreement on the research goals and more funding of enhancement research. When psychiatric researchers started to prepare the fifth edition of the *Diagnostics and Statistical Manual of Mental Disorders* (DSM) they set the aim to include biomarkers, particularly based on genetic and neuroimaging research, to improve diagnosis and treatment (Hyman, 2007). Note that the previous fourth edition of the DSM listed more than 300 disorders and their respective symptoms guiding clinical diagnosis (APA, 2000). It is now widely acknowledged that this attempt for the fifth edition was unsuccessful, though views on why this happened and what to do about it differ (Hyman, 2010; Kapur et al., 2012; Walter, 2013; Kirmayer and Crafa, 2014). Certainly, with more than one billion dollars annually spent on research at the National Institutes of Mental Health alone, lack of funding was not the problem^3^. In the light of decisions by pharmaceutical companies to close their psychiatric laboratories because of negative prospects (Amara et al., 2011; Van Gerven and Cohen, 2011) and reports that prescription stimulants do not even seem to have a lasting positive effect on individuals diagnosed with Attention Deficit/Hyperactivity Disorder (Currie et al., 2013; Sharpe, 2014), the frequently promised translational possibilities of enhancement research may be unrealistic (Schleim, 2014). Perhaps we need to minimize risks of committing a translational fallacy, too.

When Quednow speaks of a “phantom debate” (Quednow, 2010) or Lucke and colleagues want to deflate the “neuroenhancement bubble” (Lucke et al., 2011), they appear to have good reasons for doing so. We should also not forget that people in many countries are already quite happy and that in those where they are not, the difference in happiness is probably not due to limited access to enhancement technology. Clinical research for those suffering from a disorder should keep the priority over enhancement. It could even be the case that too much focus on increasing well-being and happiness, on how things might yet be better than they presently are, might make more people unhappy in the first place; or, in Schopenhauer's words:

“We then recognize that the best, which the world has to offer, is a painless, calm, bearable existence and we confine our claims to these in order to accomplish them better. Because not to become very unhappy, it is the best means that one may not demand to be very happy.” (Schopenhauer, 1874, p. 434; author's translation).

### Conflict of interest statement

The author declares that the research was conducted in the absence of any commercial or financial relationships that could be construed as a potential conflict of interest.

## Footnotes

^1^http://www.frontiersin.org/Systems_Neuroscience/researchtopics/Augmentation_of_Brain_Function/1563

^2^http://www.oecdbetterlifeindex.org (accessed May 30, 2014)

^3^http://www.nimh.nih.gov/about/budget/index.shtml (accessed May 30, 2014)
